# MeCP2 plays an analgesic role in pain transmission through regulating CREB / miR-132 pathway

**DOI:** 10.1186/s12990-015-0015-4

**Published:** 2015-04-12

**Authors:** Ran Zhang, Min Huang, Zhijuan Cao, Jieyu Qi, Zilong Qiu, Li-Yang Chiang

**Affiliations:** The Key Laboratory of Developmental Genes and Human Disease, Ministry of Education, Institute of Life Sciences, Southeast University, Nanjing, China; Institute of Neuroscience, Key Laboratory for Primate Neurobiology, Chinese Academy of Sciences, Shanghai, China; The Kellogg School of Science and Technology, the Scripps Research Institute, La Jolla, CA 92037 USA

**Keywords:** MeCP2, Acute pain, SNI model, Spinal cord, p-CREB/miR-132

## Abstract

**Background:**

The Methyl CpG binding protein 2 gene (*MeCP2* gene) encodes a critical transcriptional repressor and is widely expressed in mammalian neurons. MeCP2 plays a critical role in neuronal differentiation, neural development, and synaptic plasticity. Mutations and duplications of the human *MECP2* gene lead to severe neurodevelopmental disorders, such as Rett syndrome and autism. In this study we investigate the role of MeCP2 in the spinal cord and found that MeCP2 plays an important role as an analgesic mediator in pain circuitry.

**Findings:**

Experiments using MeCP2 transgenic mice showed that overexpression of MeCP2 weakens both acute mechanical pain and thermal pain, suggesting an analgesic role of MeCP2 in acute pain transduction. We found that through p-CREB/miR-132 signaling cascade is involved in MeCP2-mediated pain transduction. We also examined the role of MeCP2 in chronic pain formation using spared nerve injury (SNI) model. Strikingly, we found that development of neuropathic pain attenuates in MeCP2 transgenic mice comparing to wild type (WT) mice.

**Conclusions:**

Our study shows that MeCP2 plays an analgesic role in both acute pain transduction and chronic pain formation through regulating CREB-miR-132 pathway. This work provides a potential therapeutic target for neural pathologic pain, and also sheds new lights on the abnormal sensory mechanisms associated with autism spectrum orders.

## Findings

### Background

Mutations of methyl-CpG binding protein 2 (MeCP2) lead to Rett syndrome, a severe neurodevelopmental disorder [1]. Whereas, duplications of mecp2 locus lead to severe autism spectrum disorders in human patients [2]. Dysfunctions of pain sensitivity have been reported in human Rett syndrome patients [3]. Interestingly, it is recently reported that MeCP2 plays a critical role in persistent pain sensation through central mechanisms [4].

To mimic MECP2 duplication syndrome, genomic segment containing human *MECP2* gene has been introduced into the mouse genome [5]. Mice carrying human *MECP2* transgene appear to have defects in social interaction, anxiety, and motor functions [6]. Whether pain sensation would be altered in mecp2 transgenic mice or human patients with MECP2 duplication syndrome are unknown.

Peripheral nerve injury causes not only central sensitization [7], but also protection mechanism against injury. Central sensitization represents for an enhancement in the function of neurons and formation of circuits in nociceptive pathways caused by increases in membrane excitability and synaptic efficacy as well as reduced inhibition and a manifestation of the remarkable plasticity of the somatosensory nervous system in response to activity, inflammation, and neural injury [8]. Protection responses include remodeling of nervous system circuitry related to the lost functions [9] and intrinsic mechanisms of pain inhibition [10,11]. Under the condition of neuropathic pain, a wide range of functional genes need to be expressed [12,13] when protection response occurs.

MeCP2 expresses predominantly in mature neurons [14]. It acts as transcriptional repressor factor through its methyl CpG binding domain and transcriptional repressor domain [15,16]. Many studies showed that MeCP2 can also activate gene expression of a variety of genes such as brain-derived neurotrophic factor (BDNF) [17,18]. In recent studies, microRNAs attract more and more attention in post-transcriptional regulation of gene expression. MicroRNAs are short RNA transcripts with 20 ~ 25 bp that regulate gene expression through complementary pairing with 3′UTR of mRNAs thus prevent translation [19]. Studies showed that miR-132 can be induced by p-CREB [20]and take part in restricting MeCP2 level within a narrow range [21,22].

Previous study suggests that 75.2% of Rett Syndrome patients induced by MeCP2 mutation have a deficit in normal pain response [3]. In rat experiments, researchers using microarray analysis found that most genes that are regulated in inflammatory pain are either MeCP2 target genes or genes that can form transcriptional repressor compound with MeCP2 [23]. In both neuropathic pain and inflammatory pain models, it is found that MeCP2 expression or p-MeCP2 has a significant change [24,25]. All of these suggest a relationship between MeCP2 and pain sensitivity.

Our results show that MeCP2 overexpression attenuates both mechanical and thermal pain sensitivity. The mechanism is through CREB/miR-132 signaling pathway in spinal cord. This suggests a novel therapeutic approach for suppressing formation of chronic pain using MeCP2 as a target gene.

### Results

#### The role of MeCP2 in acute pain sensitivity

**Figure 1:**
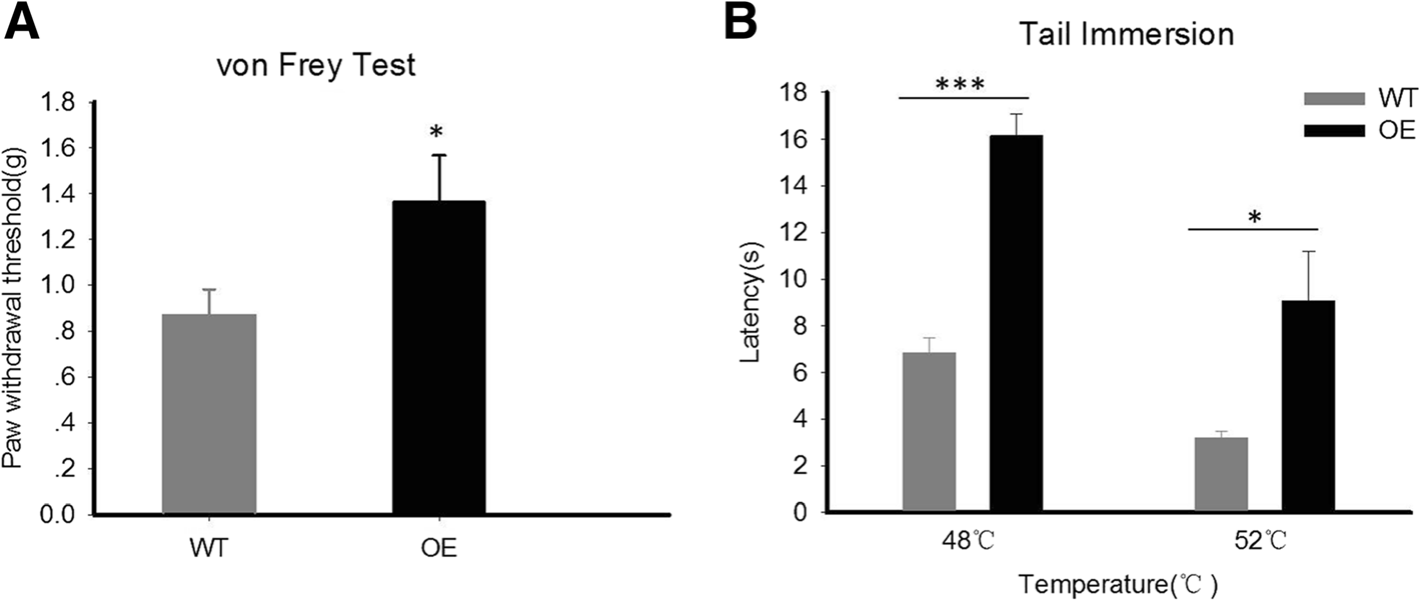
MeCP2 overexpression attenuates both mechanical and thermal pain sensitivity. **A**, Comparison of paw withdrawal threshold to mechanical stimulation between WT mice and MeCP2 overexpression mice (OE). 7 mice were tested in each group. **B**, Comparison of pain sensitivity to thermal stimulation between WT mice and MeCP2 overexpression mice (OE). n = 9 for WT and OE respectively. All values are represented as means ± SEM. *p < 0.05, **p < 0.01, ***p < 0.001.

We used wild-type (WT) and transgenic mice overexpressing MeCP2 (OE) to examine whether MeCP2 is involved in pain sensitivity. Both mechanical sensitivity and thermal sensitivity were investigated by various tests. Paw withdrawal threshold was studied using the *von Frey* test. We found that MeCP2 OE mice had a significantly higher paw withdrawal threshold than WT mice (Figure 1A). Under thermal stimulation, MeCP2 OE mice showed significantly longer latency in tail immersion than WT at both 48°C and 52°C (Figure 1B). These results suggest that MeCP2 plays a critical role in pain sensitivity modulation.

#### MeCP2 weakens pain sensitivity through central mechanism

MeCP2 expression level changes under pain condition [24,25], but the mechanism of its involvement in pain states remains unknown. Previous studies identified the ischemic stimulus that triggers an endogenous, neuroprotective response which protects the brain during a subsequent severe ischemic injury through a rapid increase in MeCP2 protein [22]. So the location of MeCP2 and how it changes under pain conditions should be investigated.

**Figure 2:**
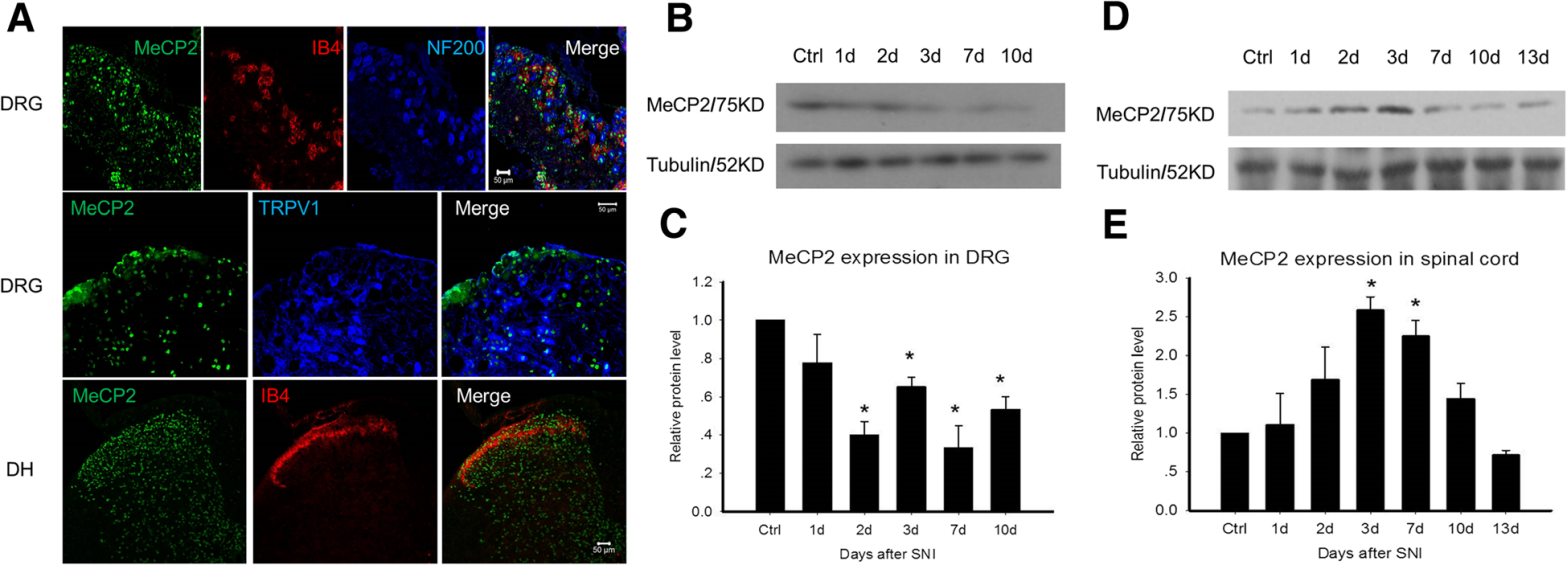
MeCP2 weakens pain sensitivity through central mechanism. **A**, The location of MeCP2 expression in DRG and spinal cord dorsal horn (DH). In DRG, all IB4 (red) positive neurons and NF200 (blue) positive neurons have MeCP2 (green) expression (upper panel). All TRPV1 (blue) positive neurons have MeCP2 (green) expression (mid panel). In DH, MeCP2 expressed more intense than other area of spinal cord (lower panel). Representative pictures show single focal plane. Scale bars, 50 μm. **B**, In DRG, MeCP2 protein level was downregulated after SNI. **C**, Relative protein levels of MeCP2 expression in DRG. **D**, In spinal cord, MeCP2 protein level was elevated by 3d to 7d after SNI, and then downregulated. **E**, Relative protein level of MeCP2 expression in spinal cord. Bars are relative protein level to control without SNI. Error bars are SEM. *p < 0.05 (t test).

In our experiments, frozen sections of DRG and spinal cord were obtained from lumbar L4-L6. Co-localization of MeCP2 with nociceptive neurons and non nociceptive neurons were investigated using immunofluorescence, which demonstrated that all pain sensitive neurons (IB4 and TRPV1 positive) as well as non nociceptive neurons (NF200 positive) displayed MeCP2 expression. This finding provided the material proof for MeCP2 taking part in pain states (Figure 2A).

The Spared nerve injury (SNI) model was used to study MeCP2 expression under pain conditions. We performed a time series of 10 days and 13 days to observe variation tendency of MeCP2 in Dorsal root ganglion (DRG) and spinal cord respectively. In DRG, MeCP2 protein level was down-regulated after SNI (Figure 2B and C). In spinal cord, MeCP2 protein level was elevated from 3d to 7d after SNI, and then down-regulated (Figure 2D and E). So MeCP2 may act as a protective factor in central system under neuropathic pain.

#### The role of MeCP2 in chronic pain

Despite considerable knowledge of the pain mechanism having been accumulated [12,13,26], there is no consummate treatment for chronic pain. Since MeCP2 is critical for pain sensitivity (Figure 1) and its upregulation for protective responses under pain condition (Figure 2D and E), there would be a new research direction if elevating MeCP2 may suppress the sense of pain.

**Figure 3:**
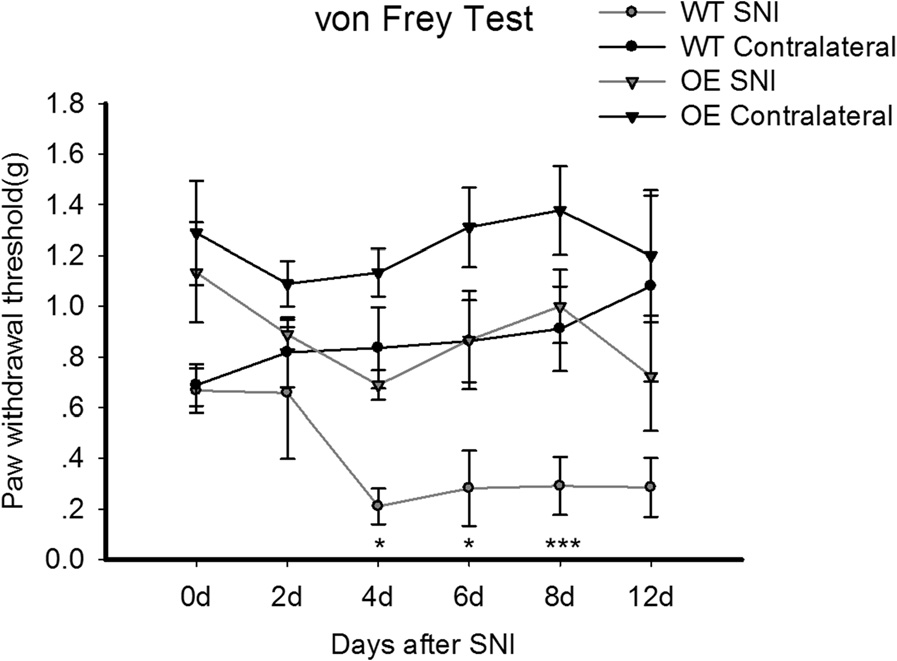
The role of MeCP2 in pain suppression. In WT mice, hindpaws after SNI showed significant reduction in withdrawal threshold compared to contralateral hindpaws in mechanical *von Frey* test. But MeCP2 overexpression mice only had a slight downgrade in paw withdrawal threshold and their withdrawal threshold after SNI shows no diffierence compared with WT contralateral. 9 mice were tested in each group. All values are represented as means ± SEM. *p < 0.05, **p < 0.01, ***p < 0.001.

We next further examine the role of MeCP2 in chronic pain using WT and MeCP2 OE mice. The right hindpaws of WT mice were regarded as control. The left hindpaws of WT mice and MeCP2 overexpression mice were followed by SNI. In *von Frey* test, hindpaws after SNI showed significant reduction in withdrawal threshold compared to contralateral hindpaws, while MeCP2 OE mice only shows a slight downgrade in paw withdrawal threshold and their withdrawal threshold after SNI had no difference compared with WT contralateral (Figure 3). These evidence strongly suggests that MeCP2 could suppress sense of pain.

#### CREB/miR-132 pathway is involved in the protective response of MeCP2

**Figure 4:**
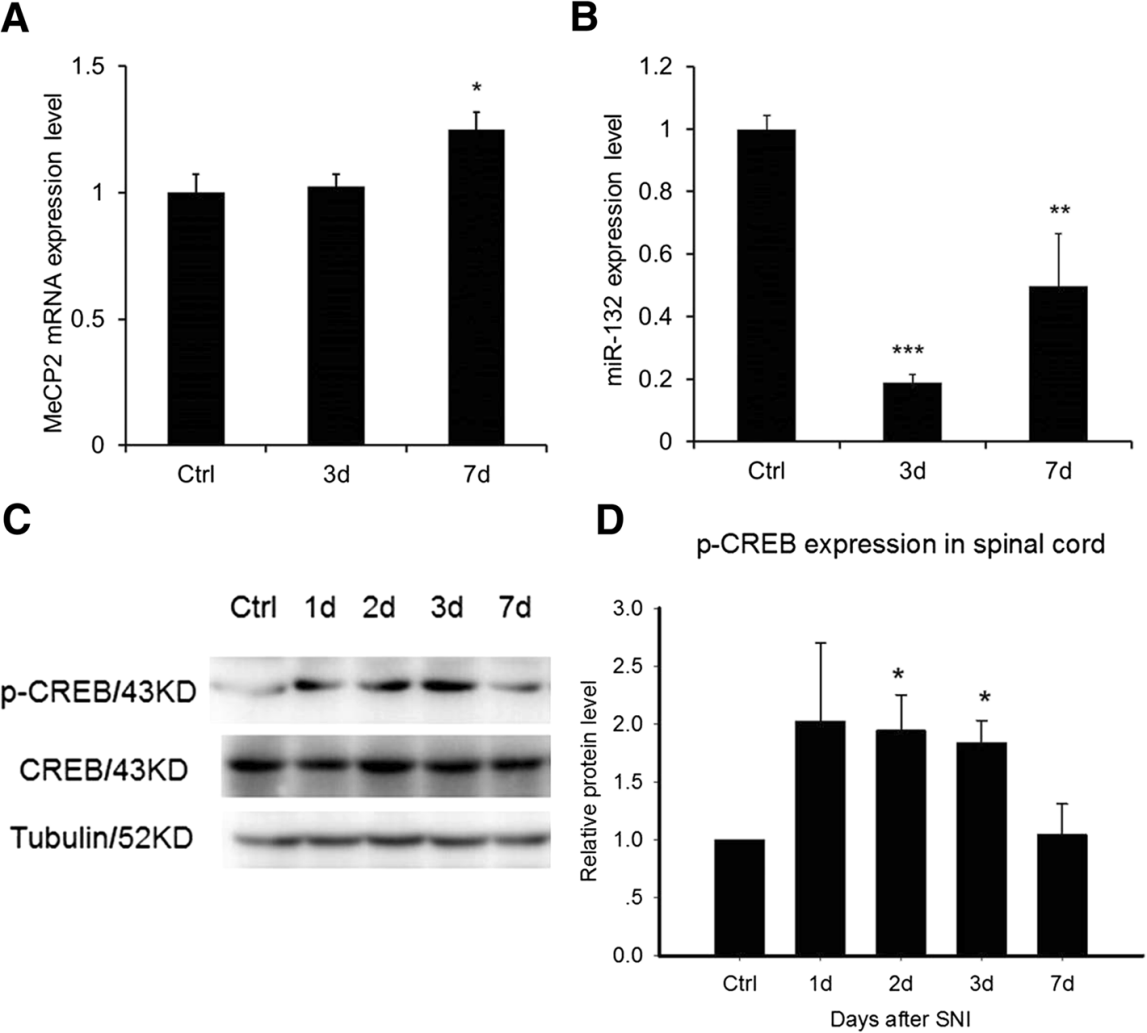
Change of MeCP2 transcript, miR-132 and p-CREB after SNI. **A**, MeCP2 transcript did not change significantly after SNI. **B**, miR-132 was downregulated by 3d to 7d after SNI. RNAs were collected from lumbar spinal cord L4-L6 lysates of WT mice. Levels of MeCP2 transcript and miR-132 were analyzed by qPCR. **C**, p-CREB(Ser133) was elevated by 3 days after SNI and then fell off. **D**, Relative protein level of p-CREB expression in spinal cord. Bars are relative protein level to control without SNI. Error bars are SEM. *p < 0.05 , **p < 0.01 (t test).

MeCP2 is generally a transcriptional repressor factor [16]. Its upregulation leads to suppression of target genes [15]. MeCP2 is also the target of others regulators [21]. Both MeCP2 mutation and overexpression will lead to neurological diseases, which suggest that MeCP2 expression in the proper range is necessary for normal physiological function. We found that mRNA level of MeCP2 did not change significantly after SNI, but the protein level was elevated (Figure 4A). So change of MeCP2 expression was at the post-transcriptional level. MicroRNAs are short RNA transcripts with 20 ~ 25 bp that regulate gene expression at the post-transcriptional level. Several studies have found that MeCP2 can be regulated by miRNAs especially miR-132 in specific areas of the brain. Spinal cord may share the similar mechanism with brain. Thus we would like to examine levels of miR-132 in SNI experiments. We found that expression of miR-132 were detected in Spinal cord after SNI (Figure 4B). Interestingly, miR-132 was downregulated by 3d to 7d after SNI (Figure 4B).

The induction and persistent stages of pain is a balanced process between pain-induction and self-repair in which new proteins needed to be synthesis. Some are “pain genes” like c-fos, a product of immediate early gene that is induced by p-CREB [27,28]. Others are “Analgesic genes”. In the induction period of neuropathic pain, the phosphorylation level of cAMP response element binding protein (p-CREB S133) was elevated rapidly to activate pain-related genes (Figure 4C and D). C-fos is one of the target genes that is induced by p-CREB. From 3 days to 7 days after SNI, the p-CREB level returned (Figure 4C and D). miR-132 that is induced by p-CREB was also downregulated (Figure 4B). miR-132 forms complementary pairing with 3′UTR of MeCP2 and prevent its expression at post-transcriptional level. MeCP2 was released to express when miR-132 decrease and provided injury body a protective response.

### Discussion

#### Mechanisms of acute and chronic pain and common treatments

Acute pain is defense mechanism that protects body from noxious harmful stimuli, which is essential to an organism’s survival and wellbeing. When physical damage occurs or short-term pain is not properly treated, acute pain can develop into chronic pain, a kind of disease that can evoke persistent pain without any noxious stimuli or enhanced sensitization to noxious stimuli, which is termed as allodynia and hyperalgesia in clinics. Chronic pain happens due to increased spontaneous firing or alterations in neurotransmitter properties at peripheral level or alterations in excitatory neurotransmitter secretion, inhibitory interneurons and glial-neuronal interactions at central level. Common treatments are centered around non-steroidal anti-inflammatory drugs (NSAIDs) and opiates and are susceptible to addiction and respiratory depression. New therapeutic strategies with efficiency and less side effects are still highly demanded. Since ReTT syndrome patients with MeCP2 mutation were found having abnormal pain sensitivity [3], targeting MeCP2 in local region, which is responsible for pain sensation but not other physiological functions will take more attention in future study.

#### Pain and memory share similar mechanisms

Synaptic plasticity is fundamental to many neurobiological functions, including learning and memory as well as pain. Synaptic strength is dynamic rather than static in response to changes in transmitter release from presynaptic terminals or response of the postsynaptic membrane. MeCP2 is found in regulating synaptic characters through its targets which responsible for synaptic plasticity [29]. Both deficiency and dulplication of MeCP2 can change density of the dendrits and morphology of dendritic spines. By studying brain areas such as hippocampus do people identified the electrophysiological properties of LTP (long-term potentiation) and morphological change of dendritic spines. LTP (long-term potentiation) and LTD (long-term depression) are highly associated with formation of learning and memory and are based on synaptic plasticity. Same mechanism is seen in pain transmission, comprising an activity-dependent, long-lasting facilitation of synaptic excitatory postsynaptic potentials in response to brief, high-frequency repeated trains of nociceptor input. This can be deemed as central sensitization under chronic pain. Researchers could pay close attention to the mechanisms of MeCP2 responsible for synaptic plasticity in the induction and maintenance of central sensitization for analgesic therapy.

#### MicroRNAs in pain signalling pathway

MicroRNAs are small, noncoding RNA molecules that direct the post-transcriptional suppression of gene expression. They have antisense complementarity to multiple sites in the 3′UTR of target genes, raising RNA-induced silencing complex (RISC) to cleave the mRNAs or prevent mRNAs from translation. MicroRNAs are involved in several developmental, physiological, and pathophysiological processes and play an important role in neurogenesis, neuron survival, dendritic outgrowth, and spine formation. Few studies have addressed the role of miRNAs in neuropathic pain. Some miRNAs link to pain-related pathophysiology while some miRNAs alleviate neuropathic pain in spinal cord. The microRNA212/132 family is one of the most studied miRNA family closely related to MeCP2. Transgenic miR-132 alters neuronal spine density and impairs novel object recognition memory. Klein et al. have proposed a homeostatic feedback mechanism that as a target of MeCP2, BDNF induces miR-132 synthesis through CREB pathway, miR-132 in turn silence MeCP2 expression [21]. Thereby, the neuroprotective response of MeCP2 induced by getting rid of miR-132 inhibition is speculated and demonstrated.

#### Study limitations and perspective

Genetic background affects nociceptive pain sensitivity in animals and may influence susceptibility to the development of persistent pain. MeCP2 transgenic mice used in experiments are systemically overexpressed in whole body. Thus its distinction in behavioral phenotypes from WT may not be confined to spinal cord. A local overexpression and knockdown of MeCP2 in spinal cord is yet to be produced for further study of the analgesia of MeCP2 at central level.

### Conclusions

MeCP2 alleviated acute pain and neuropathic pain through P-CREB/miR-132 pathway in spinal cord.

### Methods

#### Animals

FVB mice were purchased from Nanjing Biomedical Research Institute of Nanjing University. mecp2 TG mice (008679) were purchased from Jackson Laboratory. Animals were kept in their home cage at 21°C and 55% relative humidity with a 12 h light/dark cycle and were fed food and water adlibitum. All animal experiments were approved by the Animal Experiments Committee (Approval No. DWSY0121) of the Institute of Life Sciences at Southeast University in accordance with the International Association for Study of Pain regulation.

#### Surgeries

The spared nerve injury (SNI) model was used in experiments. Animals were anaesthetized with 10% chloral hydrate under intraperitoneal injection. Skin of left knees were opened and the muscle layer were separated to expose the sciatic nerve. 5-0 suture is applied around the tibial and common peroneal nerves, while the sural nerve is left to be completely intact. Then a tight surgical knot is made and a short section below the knot is cut. Next the muscle layer is gently closed and sutured. The right knees were also submitted to exposure the sciatic nerve without injury as control. Animals were recovered 2 days before behavior test.

#### Behavior tests

##### von Frey test

Mice were placed in blue colored plastic cylinders placed on a wire mesh table. Habituated for 1 hour in cylinders prior to testing. Applied *von Frey* filaments to the lateral part of the paw. Response in four out of eight stimuli is regarded as a positive reaction.

##### Tail immersion

The animals’ tails were placed in a water bath heated to 48°C and 52°C respectively, and the latency of response was measured upon tail flicking. The cut-off time of 20 s was set to prevent tail skin tissue damage. Each animal was tested for 3 times, and interval at least 10 min.

#### Immunofluorescence

Mice were deeply anaesthetized with 10% chloral hydrate. Perfusion was performed with PBS followed by 4% paraformaldehyde (PFA) in 0.1 M phosphate buffer pre-fixation. DRG or lumbar spinal cord L4-L6 were dissected for a post- fixation into 4% PFA for one day then transferred to 30% sucrose solutions for 3 days. Tissues were embedding by OCT at -20°C. Frozen sections were made in 10 μm(DRG) and 25 μm (spinal cord). Sections were washed in PBS, blocked with 10% donkey serum, permeabilized in PBS containing 0.3% Triton X-100 (PBT) for 2 h, and incubated with anti-MeCP2 antibody (CST, #3456, 1:500), anti-NF200 antibody(SIGMA, N0142, 1:250), anti-TRPV1 antibody(Millipore, MAB5568, 1:600) diluted in blocking solution at least 16h at 4°C. Sections were subsequently washed in PBS, incubated with the anti-rabbit IgG-FITC (Santa Cruz, sc-2090, 1:300), anti-mouse IgG-CFL 647 (Santa Cruz, sc-362288, 1:300) and Isolectin GS-IB4 (Invitrogen, 121412, 1:1000) for 2 h at room temperature and washed with PBS three times. As a last step, coverslips were applied.

#### Quantitative real-time PCR assays

Total RNA was extracted from mice L4-L6 spinal cord using mirVana miRNA Isolation Kit (Ambion). For the measurement of primary miRNA transcripts, the large-sized RNA fractions (>200 bp) were used for reverse transcription. cDNA was synthesized by poly-dT primers from 1 mg of purified RNA (>200 bp fraction) by iScript cDNA Synthesis Kit (Bio-Rad). SYBR Premix Ex Taq from Takara was used in this study. Quantitative real-time PCR was performed with the Rotor-Gene Q machine (QIAGEN). Results were normalized to GAPDH, and data analysis was done by using the comparative CT method in software by QIAGEN.

Primers used in quantitative real-time PCR assays were as follows:

miR-132 forward, 50-ACCGTGGCTTTCGATTGTTA-30

miR-132 reverse, 50-GGCGACCATGGCTGTAGACT-30

MeCP2 forward, ACAGCGGCGCTCCATTATC

MeCP2 reverse, CCCAGTTACCGTGAAGTCAAAA

#### Immunoblotting

For western blot analysis, total proteins were extracted from mice L4-L6 DRG and spinal cord using FDTMRIPA Buffer add protease inhibitors and phosphatase inhibitors (Roche). Protein sample was separated on a 10% polyacrylamide gel and electrotransferred to nitrocellulose membrane (Milipore). Membranes were blocked in 5% skim milk at room temperature for 1h and then incubated with primary antibody at 4°C overnight. After washing with TBST, membranes were incubated with HRP-conjugated secondary antibody at room temperature for 1h. Targeted proteins were visualized with the SuperSignal(R) West Pico Chemiluminescent Substrate (Thermo Scientific). Primary antibody were used as follows: anti-MeCP2 antibody (CST, #3456, 1:1000). anti-Tubulin antibody (CST, #2125, 1:1000). anti-CREB antibody (CST, #9197, 1:1000). anti-p-CREB antibody (Santa Cruz, sc7978, 1:200). Secondary antibody were used as follows: Goat anti-rabbit IgG-HRP (ABmart). Donkey anti-goat IgG –HRP (ABclonal).

#### Statistical analysis

All the data were analyzed with Student’s t test, and P values of less than 0.05 were regarded as statistically significant (indicated with an asterisk in summary graphs). Values were presented as means ± standard errors of the means (SEM).
